# The activation of EGFR promotes myocardial tumor necrosis factor-α production and cardiac failure in endotoxemia

**DOI:** 10.18632/oncotarget.6071

**Published:** 2015-10-10

**Authors:** Xuegang Sun, Jiani Liang, Xueqing Yao, Chunhua Lu, Tianyu Zhong, Xiaoyang Hong, Xiaofei Wang, Wenjuan Xu, Miaoning Gu, Jing Tang

**Affiliations:** ^1^ The Department of Anesthesia, Nanfang Hospital, Southern Medical University, Guangzhou, Guangdong, China; ^2^ The Key Laboratory of Molecular Biology, State Administration of Traditional Chinese Medicine, School of Traditional Chinese Medicine, Southern Medical University, Guangzhou, Guangdong, China; ^3^ The Department of General Surgery, Guangdong General Hospital, Guangdong Academy of Medical Science, Guangzhou, Guangdong, China; ^4^ The Department of Laboratory Medicine, First Affiliated Hospital of Ganna Medical University, Ganzhou, Jiangxi, China; ^5^ The Department of Intensive Care Unit, BaYi Children's Hospital, Beijing Military General Hospital, Beijing, China

**Keywords:** sepsis, EGFR, tumor necrosis factor-alpha, cardiac failure, Pathology Section

## Abstract

To study the effect of EGFR activation on the generation of TNF-α and the occurrence of cardiac dysfuncetion during sepsis, PD168393 and erlotinib (both are EGFR inhibitors) were applied to decreased the production of TNF-α and phosphrylation of ERK1/2 and p38 induced by LPS in cardiomyocytes. These results were further proved by specifically knocked down the expression of EGFR *in vitro*. Both TAPI-1, a TNF-α converting enzyme (TACE) inhibitor, and TGF-α neutralizing antibody could inhibit the activation of EGFR and the generation of TNF-α mRNA after LPS treatment. The increase of TGF-α in response to LPS could also be suppressed by TAPI-1. On the other hand, exogenous TGF-α increased the expression of TNF-α mRNA and partially reversed the inhibitory effect of TAPI-1 on expression of TNF-α mRNA in response to LPS indicating that the transactivation of EGFR by LPS in cardiomyocytes needs the help of TACE and TGF-α. In endotoxemic mice, inhibition the activation of EGFR not only decreased TNF-α production in the myocardium but also improved left ventricular pump function and ameliorated cardiac dysfunction and ultimately improved survival rate. All these results provided a new insight of how EGFR regulation the production of TNF-α in cardiomyocytes and a potential new target for the treatment of cardiac dysfunction in sepsis.

## INTRODUCTION

Sepsis is a major consequence of infectious diseases triggered by Gram-positive and/or -negative organisms, which can proliferate and/or release endotoxin and lead to tissue injury or even multiple organ dysfunction syndrome [1, 2]. Until now, sepsis is still a leading cause of death in intensive care unit, however, unlike other major epidemic illnesses, treatment for sepsis is nonspecific, limited primarily to support of organ function and administration of intravenous fluids, antibiotics, and oxygen [3, 4]. In sepsis, excessive released inflammatory mediators render septic patients at high risk of developing multi-organ failure, which is associated with high mortality. Of them, heart is one of the most frequently affected organs in sepsis. Approximately 50% of the patients who are diagnosed with sepsis exhibit signs of myocardial dysfunction [5]. Mortality in septic shock or severe sepsis with cardiac dysfunction is 40-80% [6]. Endotoxins or lipopolysaccharides (LPSs) of Gram-negative bacteria are important pathogens responsible for myocardial depression [7, 8]. After binding to its innate immunity pattern recognition Toll-like receptor 4 (TLR4) [9, 10], LPS can trigger the release of many inflammatory cytokines, such as tumor necrosis factor-α (TNF-α), interleukin (IL)-1, IL-6, and IL-8 [11-13]. LPS induced TNF-α has been shown to be a major factor responsible for myocardial depression during endotoxemia and cardiomyocytes are the major local source of TNF-α [14, 15]. Therefore, TNF-α has been regarded as the important target for the treatment of endotoxemia or sepsis. So far LPS/TLR4/mitogen-activated protein kinase (MAPK)/nuclear factor-kappa B (NF-κB)/TNF-α pathway is still thought to be the classical signal pathway for production of TNF-α induced by LPS. However, in recent years, LPS was reported to transactive epithelial growth factor receptor (EGFR) [16-19]. EGFR belongs to tyrosine kinase receptor family, which is expressed in a variety of cells and plays an important role in cellular proliferation, differentiation and tumor growth [20]. In some chronic airway diseases, LPS-induced airway inflammation increases the expression of inflammatory cytokines such as IL-1, IL-6 and this effect depends on the activation of EGFR [21, 22]. Meanwhile, Küper C, et al found that in renal collecting duct cells, LPS induced EGFR activation via TLR4/TACE, and finally resulted in induction of cyclooxygenase (COX)-2 expression [23]. All these studies suggested that EGFR activation may be important in LPS induces endotoxemia. So far, there is no study especially focusing on the effect of EGFR on the production of TNF-α in cardiomyocytes and the change of cardiac function in response to LPS.

In this study, we demonstrated activation of EGFR is the key step for the production of TNF-α induced by LPS. TACE and TGF-α are needed for LPS to transactivate EGFR in cardiomyocytes. Inhibition the activation of EGFR by erlotinib can effectively alleviate cardiac disfunction and improve survival during acute endotoxemia in mice.

## RESULTS

### PD168393 and Erlotinib effectively inhibit the phosphorylation of EGFR induced by LPS

**Figure 1:**
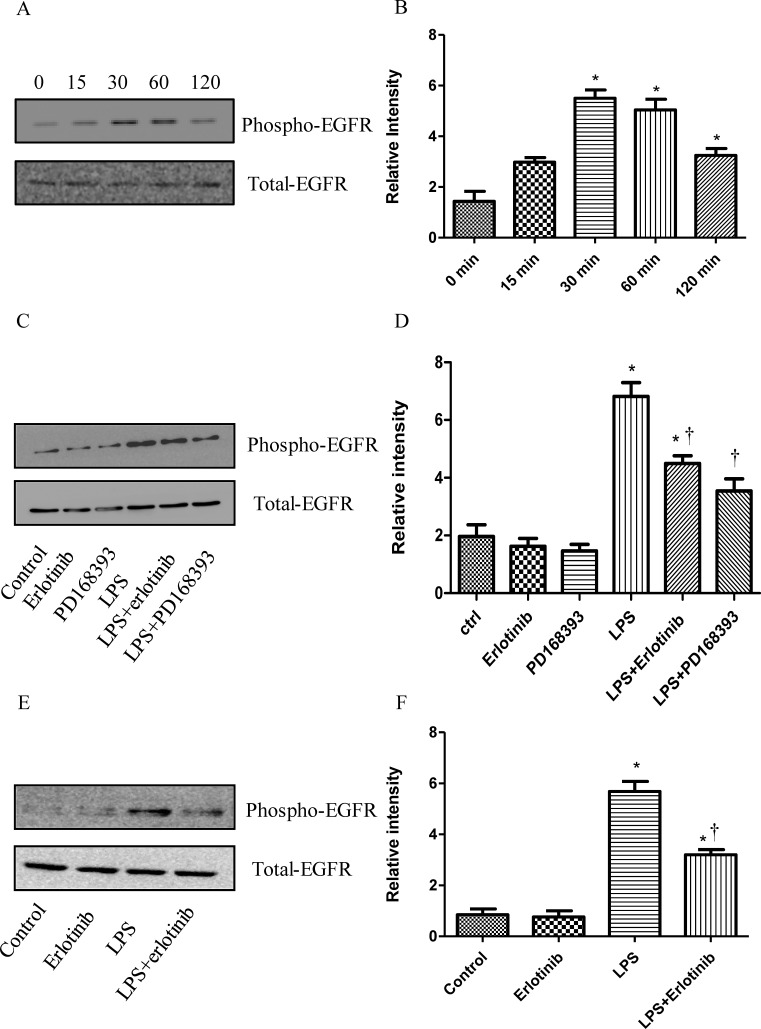
The inhibitory effect of PD168393 or Erlotinib on the trans-activation of EGFR by LPS Cardiomyocytes were pretreated with LPS (4 μg/ml). Phospho-EGFR and total EGFR were determined by western blot analysis at 0, 15min, 30min, 60min, and 120min after LPS treatment **A.**. Correspondingly gray intensity analysis of the western blot results of five groups **B.**. Cardiomyocytes were pretreated with vehicle, PD168393 (10 μM), or Erlotinib (20 μM) 0.5 hour before LPS (4 μg/ml) treatment. Phospho-EGFR and total EGFR were determined by western blot analysis at 0.5 hour after LPS treatment **C.**. Correspondingly gray intensity analysis of the western blot results of six groups **D.**. Wild type C57BL/6 mice were pretreated with erlotinib orally 3 days before LPS (5mg/kg) treatment. Phospho-EGFR and total EGFR in the myocardium were determined by western blot analysis at 1 hour after LPS treatment **E.**. Correspondingly gray intensity analysis of the western blot results of four groups **F.**. Each bar represents the mean ± S.D, *p < 0.05, compared with control group; †*p* < 0.05, compared with LPS group *n* = 4.

Although some studies have demonstrated that EGFR could trans-activate EGFR in LPS treatment [16-19], So far, there is no study focusing on the effect of LPS on the activation of EGFR in cardiomyocytes. To determine the effect of LPS on EGFR phosphorylation, we measured the phosphorylation of EGFR at 0-120 minutes after LPS treatment in cardiomyocytes. EGFR phosphorylation increased at 30 min and 60 min after LPS (4 μg/ml) treatment (Figure 1A). Further we found the trasactivation of EGFR in response to LPS could be effectively inhibited by either EGFR irreversible inhibitor PD168393 (10 μM) or reversible inhibitor erlotinib (20 μM) (Figure 1C). To further verify this result *in vivo*, 16 wild type C57BL/6 mice were divided into four groups: control group, Erlotinib group, LPS group and LPS + Erlotinib group. As shown in Figure 1E, EGFR in the myocardium was transactivated by LPS and this effect was partly inhibited by erlotinib pretreatment. All these results indicated that both *in vitro* and *vivo*, LPS induced EGFR activation can be inhibited by EGFR selective inhibitor PD168393 or Erlotinib.

### EGFR activation is required in the production of myocardial TNF-α induced by LPS

**Figure 2:**
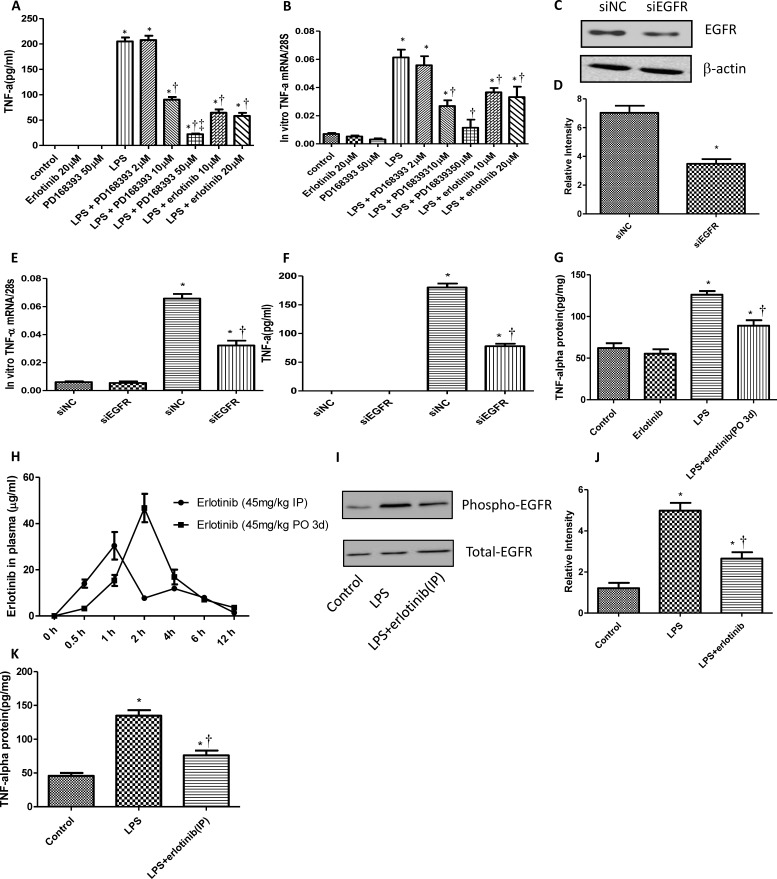
EGFR increases the production of myocardial TNF-α in response to LPS Cardiomyocytes were pretreated with vehicle, and different concentration of PD168393 or Erlotinib 0.5 hour before LPS (4 μg/ml) treatment. TNF-α protein in the cultured medium were determined by ELISA at 4 hours after LPS treatment **A.** and mRNA were measured by real-time RT-PCR at 2 hours after LPS treatment **B.**. Neonatal cardiomyocytes were transfected with si-NC and si-EGFR for 24 hrs. EGFR protein was measured by Western blot analysis **C.**-**D.** Then, the cells transfected with si-NC or si-EGFR were treated with LPS (4 μg/ml) for 2 or 4 hours to measure the expression of TNF-α mRNA expression **E.** or protein expression **F.**. Wild type C57BL/6 mice were pretreated with erlotinib orally 3 days before LPS (5mg/kg) treatment. TNF-α protein in the myocardium was measured by ELISA at 6 hour after LPS treatment **G.**. C57BL/6 mice were randomly divided into two groups: erlotinib (45 mg/kg p.o. 3d) group and erlotinib (45 mg/kg i.p.) group. The concentration of erlotinib in plasma of mice were measured at 0.5, 1, 2, 4, 6 and 12 h post-dose **H.**. Wild type C57BL/6 mice were treated with erlotinib (45 mg/kg) once through i.p. at the same time with LPS (5mg/kg). Phospho-EGFR and total EGFR in the myocardium were determined by western blot analysis at 1 hour after LPS treatment **I.**-**J.** TNF-α protein in the myocardium was measured by ELISA at 6 hour after LPS treatment **K.** Each bar represents the mean ± S.D, *p < 0.05, compared with control group; †*p* < 0.05, compared with LPS group, ‡*p* < 0.05, compared with LPS+PD168393 10μM group *n* = 4).

TNF-α is a major pro-inflammatory cytokine responsible for multi-organ failure during endotoxemia or sepsis [15]. Since LPS can transactivate EGFR in cardiomyocytes, to investigate the role of EGFR on LPS induced TNF-α expression, neonatal cardiomyocytes were pretreated with PD168393 or erlotinib 30 min before LPS (4 μg/ml) treatment. As we expected PD168393 or erlotinib obviously inhibited the production of TNF-α both in mRNA and protein level compared with LPS group. Meanwhile, as we increased the concentration of PD168393, the amount of TNF-α in the medium of cultured neonatal cardiomyocytes decreased correspondingly (Figure 2A-2B). To further verify the role of EGFR in myocardial TNF-α expression, we specially knock down the expression of EGFR in neonatal cardiomyocytes by si-EGFR technology. As shown in Figure 2C-2D, EGFR protein expression was decreased by 58% after EGFR siRNA treatment. The inhibition of EGFR expression was associated with decreased TNF-α mRNA and protein levels (Figure 2E and 2F). To verified these results *in vivo*, wild type C57BL/6 mice were treated with saline or LPS (5mg/kg, i.p.) with or without erlotinib pretreatment. Compared with LPS group, the expression of TNF-α in the myocardium of LPS + erlotinib group was effectively decreased (Figure 2G). In the LPS + erlotinib group, the mice were pretreated with erlotinib through intragastric administration for three days. We just wonder how about mice were treated with erlotinib only once just before LPS administration. As shown in Figure 2H, compared with erlotinib (45mg/kg p.o. 3d) group, the concentration of erlotinib in plasma through intraperitoneal injection rose quicker and reached to peak efficiency at 1 hour after injection. So we chose intraperitoneal injection for erlotinib treatment only once just the same time with LPS injection. By this way, erlotinib also effectively inhibited the phosphorylation of EGFR and the production of myocardial TNF-α in response to LPS (Figure 2I-2K). Allthese data suggest that the activation of EGFR promotes cardiac TNF-α expression in response to LPS.

### Inhibiting the phosphorylation of EGFR alleviates myocardial dysfunction in endotoxemic mice

**Figure 3:**
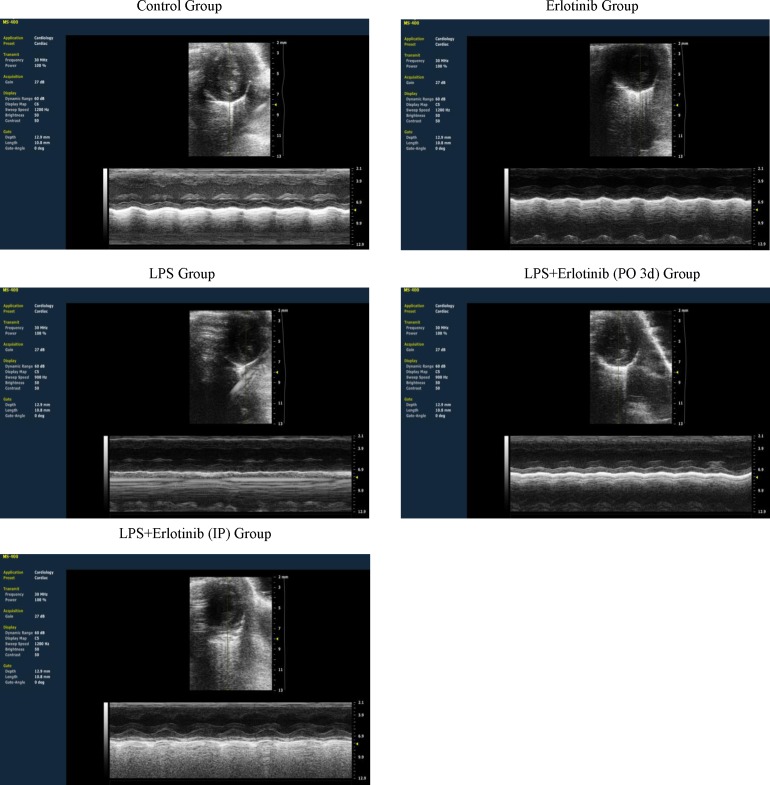
The representative left ventricle section view of cardiac ultrasound in each group

**Figure 4:**
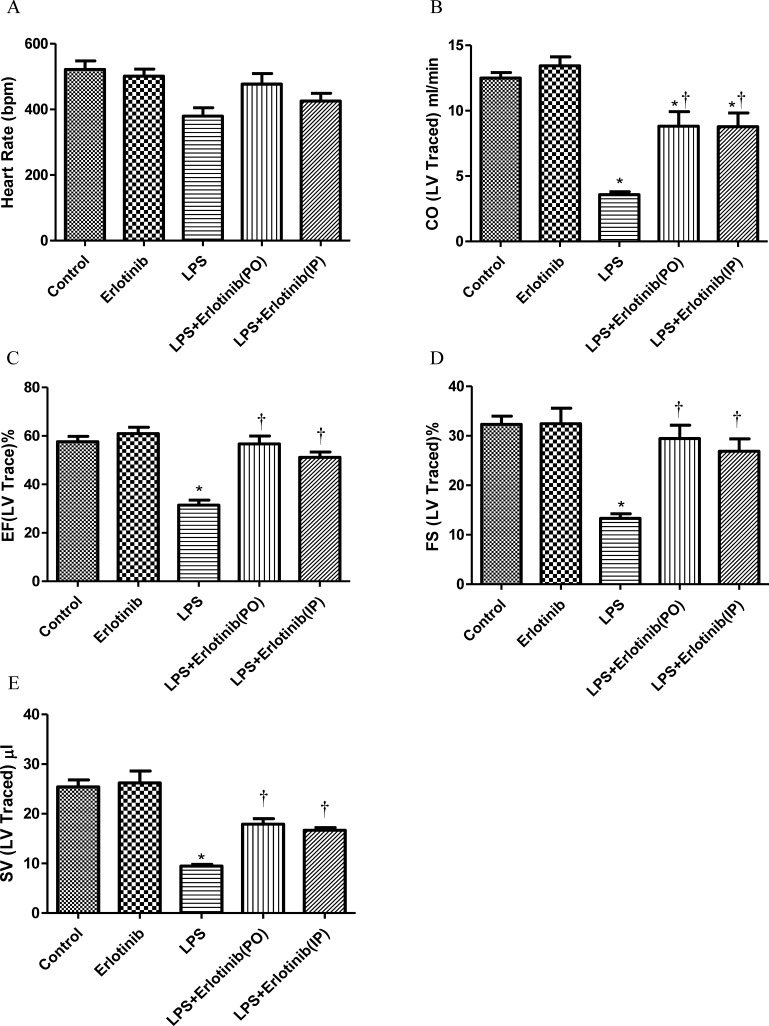
Measurement of left ventricle pump function with cardiac ultrasound during endotoxemia Wild type C57BL/6 mice were pretreated with erlotinib orally 3 days before LPS (20mg/kg) treatment or mice were treated with erlotinib through intraperitoneal injection at the same time with LPS (20mg/kg) treatment. Changes of cardiac output (CO), ejection fraction (EF), fractionalshortening (FS) and stroke volume (SV) in left ventricle were measured with cardiac ultrasound 6 hours after LPS treatment. Each bar represents the mean ± S.D, *p < 0.05, compared with control group; †*p* < 0.05, compared with LPS group, *n* = 6.

**Figure 5:**
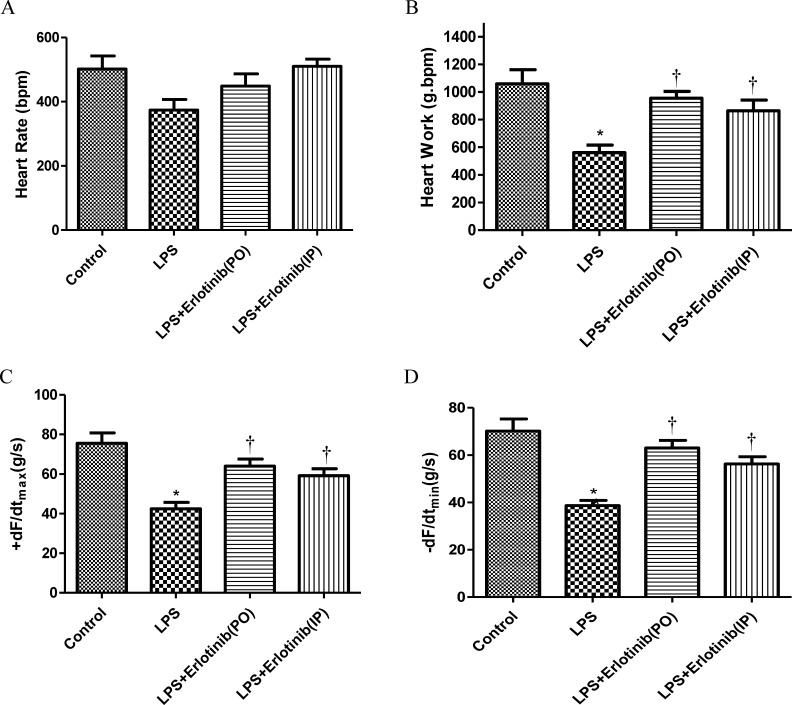
Cardiac function in mice after 6 h of in vivo LPS treatment Wild type C57BL/6 mice were pretreated with vehicle, erlotinib orally 3 days before LPS (20mg/kg) treatment or mice were treated with erlotinib through intraperitoneal injection the same time with LPS (20mg/kg) treatment. Mice hearts were isolated and perfused using the Langendorff system. Changes in heart rate **A.**, heart work **C.**, contraction (+dF/dtmax, C), and relaxation (−dF/dtmin, D) are presented. Each bar represents the mean S.D, *p < 0.05, compared with control group; † *p* < 0.05, compared with LPS group *n* = 6.

As TNF-α is one of the major factors which are responsible for the cardiac injury and failure during endotomexia or sepsis [15] and we have demonstrated that EGFR activation is crucial for cardiac TNF-α expression induced by LPS. Therefore, we further investigate the effect of EGFR activation on the hemodynamic changes of heart in endotoxemic mice with or without erlotinib treatment (45mg/kg p.o. 3d or i.p. once). Although there was no significant change of heart rate in all the five groups, the cardiac output (CO), ejection fraction (EF), fractionalshortening (FS) and stroke volume (SV) of left ventricle were significantly reduced in endotoxemic mice compared with control and erlotinib group. However all these changes induced by LPS could be obviously reversed by erlotinib both treatment and pretreatment (Figures 3-4). To avoid systemic reflex influences, we also assessed cardiac function in isolated hearts by ligandorff system. Our data demonstrated that after 6 h of LPS *in vivo* treatment, although there was no change in heart rate, the rate of contraction and relaxation and heart work were significantly reduced compared with control group. Erlotinib improved heart work and rate of contraction and relaxation in endotoxemic mice before and after treatment with LPS (Figure 5). These data demonstrated that inhibiting the phosphorylation of EGFR effectively improves left ventricular pump function and ameliorates cardiac dysfunction induced by LPS in mice.

### LPS transctivated EGFR promotes the phosphorylation of ERK1/2 and p38

**Figure 6:**
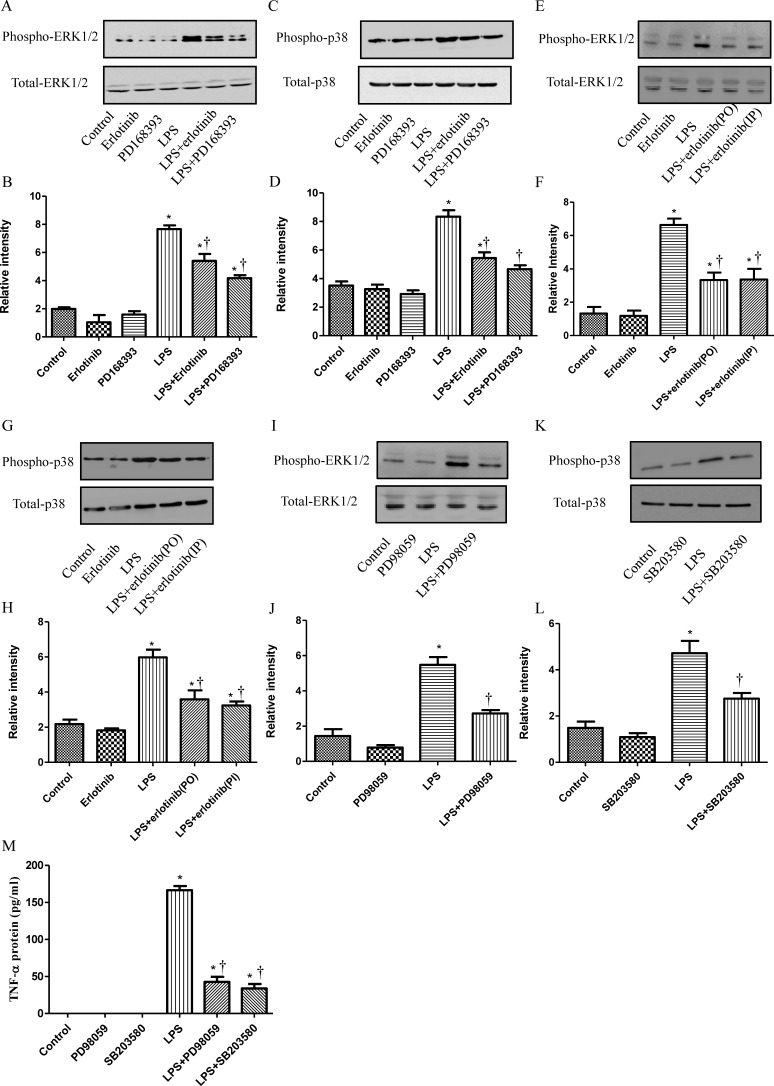
EGFR is required in the activation of p38 and ERK1/2 in LPS treated cardiomuocytes Cardiomyocytes were pretreated with vehicle, PD168393 (10 μM), or Erlotinib (20 μM) 0.5 hour before LPS (4 μg/ml) treatment. Phospho-p38 or ERK1/2 and total p38 or ERK1/2 was determined by western blot analysis at 2 hours after LPS treatment **A.**, **C.** Correspondingly gray intensity analysis of the western blot results of six groups **B.**, **D.** Wild type C57BL/6 mice were pretreated with erlotinib orally 3 days before LPS (5 mg/kg) treatment or mice were treated with erlotinib once through intraperitoneal injection the same time with LPS (5 mg/kg) treatment. Phospho-p38 or ERK1/2 and total p38 or ERK1/2 in the myocardium was determined by western blot analysis at 2 hours after LPS treatment **E.**, **G.**. Correspondingly gray intensity analysis of the western blot results of four groups **F.**, **H.** Cardiomyocytes were pretreated with PD98059 (20 μmol/L) or SB203580 (10 μmol/L) at 0.5h before LPS (4 μg/ml) treatment. Phospho-p38 or ERK1/2 and total p38 or ERK1/2 was determined by western blot analysis at 2 hours after LPS treatment **I.**, **K.** Correspondingly gray intensity analysis of the western blot results of four groups **J.**, **L.** TNF-α protein was measured in culture medium at 6h after LPS treatment **M.** Each bar represents the mean ± S.D, *p < 0.05, compared with control group; † *p* < 0.05, compared with LPS group *n* = 4.

MAPKs are the key transducers for the production of TNF-α in endotoxemia or sepsis [15, 24]. Since our results indicated EGFR activation could increase the production of TNF-α, we just wondered whether MAPKs were also involved in this signal transduction pathway. In cultured neonatal cardiomyocytes, p38 and ERK1/2 phosphorylation were measured 1 hour after LPS treatment with or without PD168393/Erlotinib pretreatment. As shown in Figure 6A-6D, LPS promoted the phosphoralation of ERK1/2 and p38 and this effect could be inhibited by EGFR selective inhibitor PD168393 or Erlotinib. Then, we verified this result *in vivo*. Wild type C57BL/6 mice were divided into five groups: control group, erlotinib group, LPS group, LPS + erlotinib (p.o. 3d) group and LPS + erlotinib (i.p.) group. Phosphorylation of ERK1//2 and p38 in the myocardium of LPS group obviously increased, compared with those of control and Erlotinib groups. Erlotinib either pretreatment or treatment at the same time with LPS could partially decrease the phosphorylation of both ERK1/2 and p38 induced by LPS (Figure 6E-6H). Then, p38 inhibitor SB203580 and ERK1/2 inhibitor PD98059 were applied to block the phosphorylation of p38 or ERK1/2 induced by LPS in neonatal cardiomyocytes. As shown in Figure 6I-6M, both SB203580 and PD98059 abrogated TNF-α expression in LPS-stimulated cardiomyocytes respectively. These results demonstrated that both p38 and ERK1/2 were involved in the mechanism of how EGFR regulating the production of TNF-α after LPS treatment.

### TACE and TGF-α are required for LPS to transactivate EGFR

**Figure 7:**
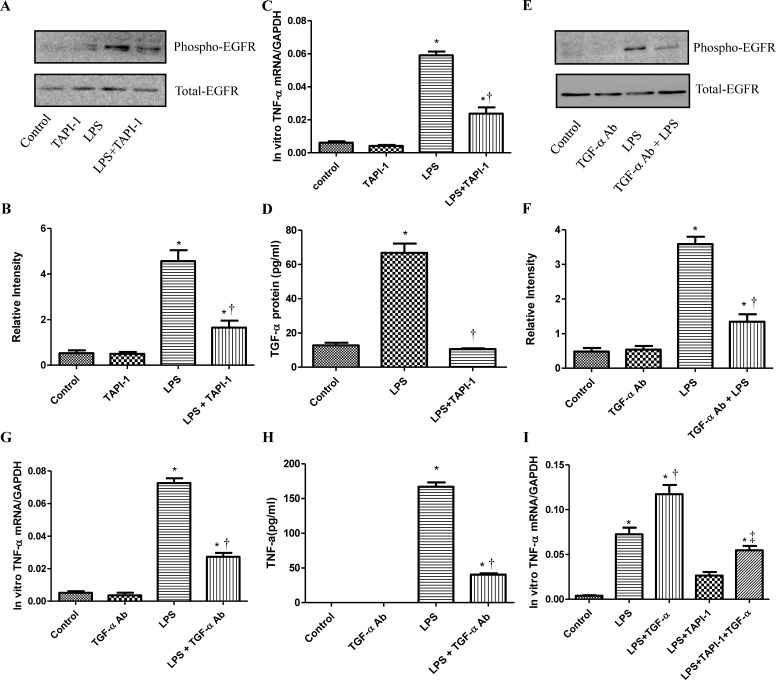
The role of TACE and TGF-α for the transactivation of EGFR and generation of TNF-α induced by LPS in cardiomyocytes Cardiomyocytes were pretreated with vehicle or TAPI-1 (30 μM) 0.5 hour before LPS (4 μg/ml) treatment. Phospho-EGFR and total EGFR were determined by western blot analysis at 0.5 hour after LPS treatment **A.**-**B.** and TNF-α mRNA was measured 2 hours after LPS treatment **C.**. Cardiomyocytes were pretreated with anti-EGFR neutralizing Ab (10 μg/ml) for 30 min to block EGFR-ligand-binding sites and then with TAPI-1 (30 μM). TGF-α in the cell culture was assayed by ELISA **D.**. Phospho-EGFR and total EGFR were determined at 0.5 hour after LPS treatment **E.**-**F.** TNF-α mRNA or protein was measured at 2 or 4 hours after LPS treatment **G.**-**H.** Cardiomyocytes were treated with TGF-α (10ng/ml) at the same time with LPS with or without TAPI-1 (30 μM) pretreatment. TNF-α mRNA was measured 2 hours after LPS treatment **I.**. Each bar represents the mean ± S.D, *p < 0.05, compared with control group; † *p* < 0.05, compared with LPS group *n* = 4.

**Figure 8:**
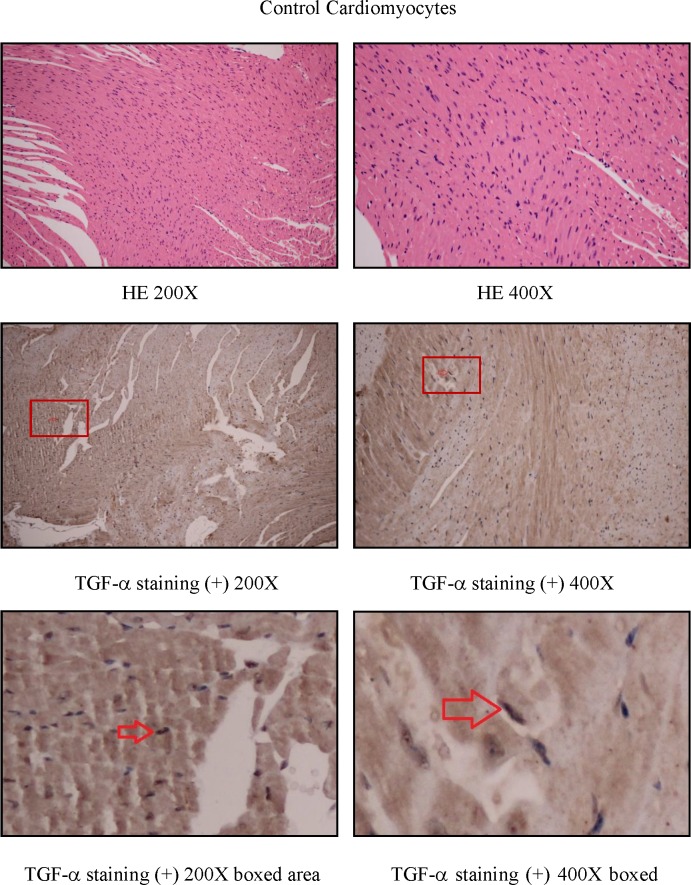
TGF-staining in left ventricle of control C57BL/6 mice

**Figure 9:**
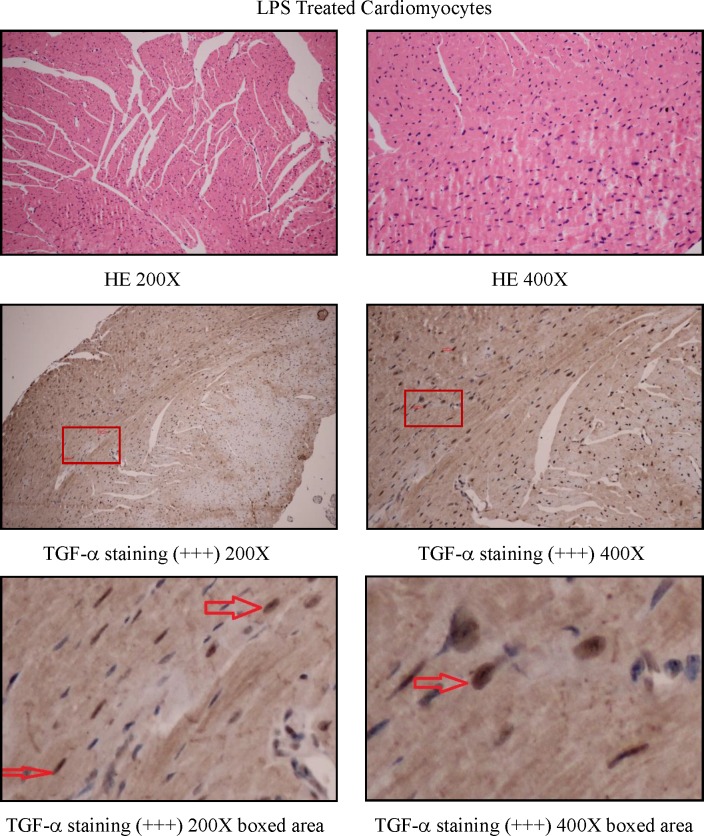
TGF-α staining in left ventricle of LPS treated C57BL/6 mice

**Figure 10:**
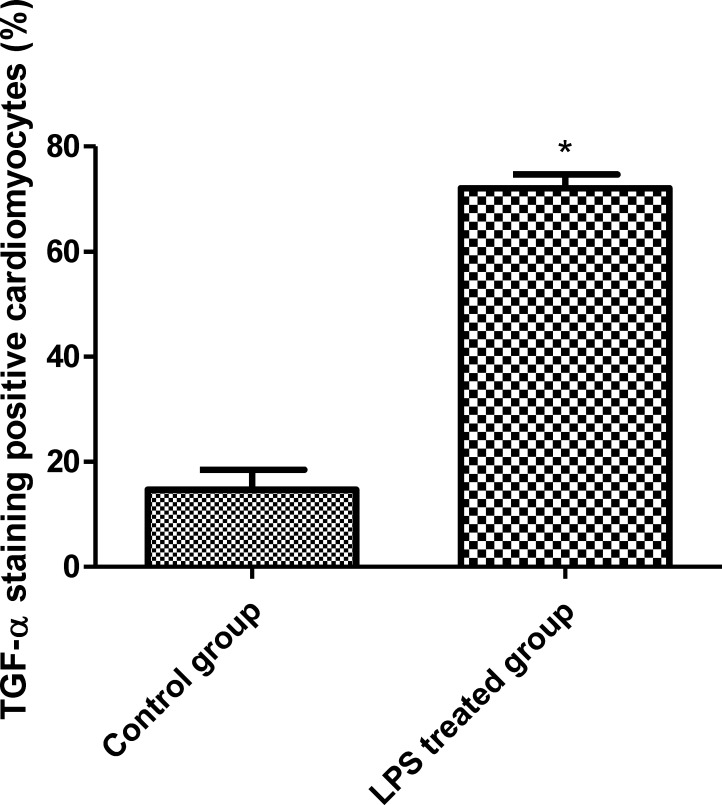
The comparison of TGF-α staining positive cardiomyocytes between the left ventricle of control and LPS treated C57BL/6 mice

To study how LPS transactivates EGFR in cardiomyocytes, TAPI-1 was used to inhibit the activity of TACE in response to LPS. As shown in Figure 7A, LPS induced EGFR phosphorylation could be effectively inhibited by TAPI-1, so did the expression of TNF-α mRNA (Figure 7C). TACE has been reported to be responsible for the ectodomain shedding of TGF-α which is a ligand for EGFR [25]. Therefore, we measured the amount of TGF-α protein in the medium of neonatal cardiomyocytes, 30 min after LPS treatment. By ELISA analysis, we found that the amount of TGF-protein was obviously increased at 30 min after LPS treatmentand and this effect could be inhibited by TAPI-1 (Figure 7D). When cardiomyocytes were pretreated with TGF-α neutralizing antibodies, the increases of TNF-α mRNA and protein production and EGFR phosphorylation in response to LPS were obviously inhibited (Figure 7E-7H). On the other hand, compared with LPS treated alone group, the expression of TNF-α mRNA was obviously increased when cardiomyocytes were treated with both LPS and TGF-α protein together. Meanwhile, the inhibitory of TAPI-1 on the expression of TNF-α mRNA in response to LPS could also be reversed by TGF-α protein (Figure 7I). To further prove the increased TGF-α levels in response to LPS derived from cardiomyocytes *in vivo*, we stained TGF-α in the myocardium of mice with or without LPS treatment for 2 h. As shown in Figures 8, 9, 10, in LPS treated left ventricle samples, the percentage of average positive TGF-α staining cardiomyocytes is about 74% or so, which is significantly higher than that of normal left ventricle samples (*P* < 0.01). All these results indicated that TACE and TGF-α are both critical important for subsequent EGFR phosphorylation and TNF-α production in response to LPS in neonatal cardiomyocytes.

### Effects of EGFR activation on the survival rate of wild type C57BL/6 mice during endotoxemia

**Figure 11:**
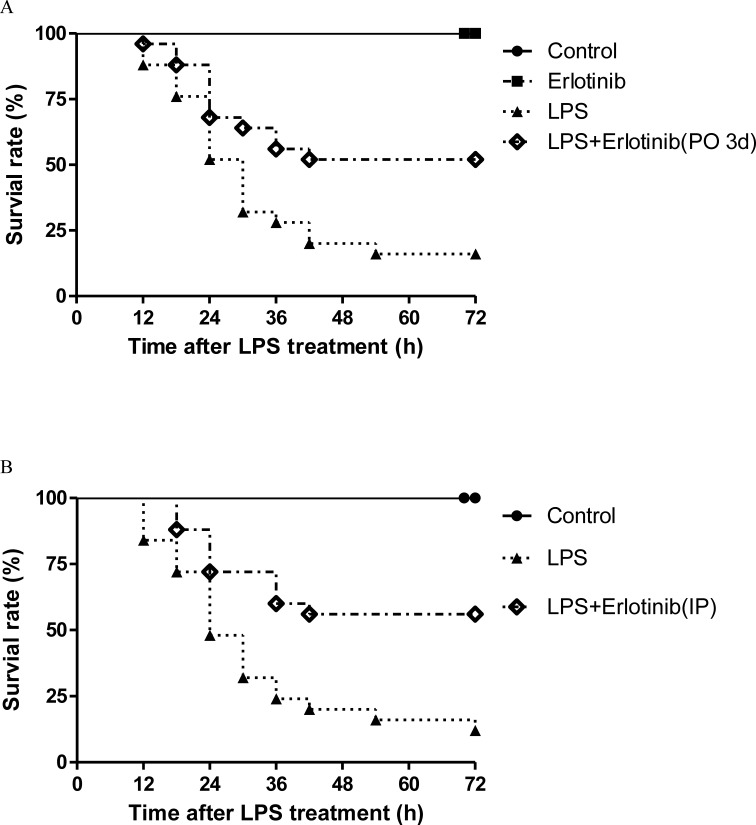
Effects of erlotinib on survival of mice treated with LPS Wild type C57BL/6 mice were pretreated with erlotinib orally 3 days before LPS (20mg/kg) treatment as described in methods **A.**. Wild type C57BL/6 mice were treated with erlotinib (45mg/kg IP) only once at the same time with LPS (20mg/kg) treatment as described in methods **B.**. After LPS stimulation, survival of mice was monitored at every 6 hour for 72 hour. Survival was significantly increased in LPS+erlotinib group (*n* = 25) compared to LPS group (*n* = 25, *p* < 0.05).

Since cardiovascular failure is the major reason responsible for the death of endotoxemic of septic mice [26], the survival rate of control, erlotinib alone, LPS and LPS+erlotinib (p.o. 3d) group were studied. LPS-treated (20 mg/kg, i.p.) mice showed signs of sepsis such as fur ruffling, conjunctivitis, and diarrhea. As shown in Figure 11A, 24 h after LPS treatment, 48% of saline-pretreated mice died, while 32% of Erlotinib-pretreated mice died and no deaths occurred in saline or erlotinib control group. At 72 h, the LPS group was associated with a 72 h survival rate of about 16%. In contrast, LPS-injected mice pretreated with erlotinib had a higher survival rate of 52%. Therefore, pretreatment with EGFR inhibitor erlotinib for three dayscould significantly improvedsurvival during acute endotoxemia in mice (*P* < 0.01). To further investigate the acute therapeutic effect of erlotinib on sepsis, we treated mice with erlotinib (45mg/kg i.p.) only once the same time with LPS administration. Compared with the LPS group, erlotinib also increased the survival rate of endotoxemic mice from 12% to 56% at 72 h after LPS treatment (Figure 11B).

## DISCUSSION

This study presented new evidence that in wild type endotoxemic mice, inhibiting the phosphorylation of EGFR can obviously attenuate cardiomyocytes produced TNF-α via EGFR/p38/ERK1/2 signal pathway. As a consequence, EGFR reversible inhibitor, erlotinib reversed LPS-induced left ventricle depression and improved survival in acute endotoxemic mice. In neonatal cardiomyocytes, both TACE inhibitor TAPI-1 and TGF-α neutralizing antibody effectively inhibited EGFR phosphorylation and TNF-α production. Furthermore, exogenous TGF-α protein could promote the expression of TNF-α mRNA and alleviated the inhibitory effect of TAPI-1 on TNF-α mRNA expression in response to LPS. All these results indicated that LPS-TLR4/TACE/TGF-α/EGFR/MAPKs represented a novel signal pathway by which LPS stimulates TNF-α production in cardiomyocytes and EGFR might be a potential therapeutic target for the treatment of endotoxemia or sepsis.

Sepsis or endotoxemia is characterized by a cascade of events that can evolve from multiorgan dysfunction to failure and death [27]. There is a high mortality rate associated with sepsis, especially for severe sepsis, defined by the presence of acute organ dysfunction, which is the leading cause of death in intensive care units (ICUs) [28, 29]. In sepsis, heart is one of the most frequently affected organs and patients, who develop myocardial dysfunction are more likely to die compared with those without evidence of myocardial dysfunction [30]. The inhibitory effect of LPS or endotoxin on cardiac function is mediated through the generation of inflammatory cytokines. Of them, TNF-α is a major cytokine involved in promoting cardiac dysfunction during sepsis [7, 31, 32]. By whole-body excision of LPS induced TNF-α in a mouse model, Merrill JC, et al. demonstrated that both systemic and local forms of inflammation are significantly reduced [33], which seemed to shed a new light on the treatment of sepsis. Howerer, until now, the success is very limited in clinical trials, although many efforts had been made to therapeutically inhibit TNF-α [34]. All these results indicated that we need to find some new targets to decrease the excessive production of TNF-α not only just focusing on the anti-TNF-α agents.

EGFR (also known as HER1) belongs to the ERBB family of cell-surface receptor tyrosine kinases which also includes HER2 [35]. After binding to its ligands, EGFR triggers homodimerization or heterodimerization of this receptor with other ERBB members, namely HER2, and activates downstream effectors such as RAS-RAF-MEK-ERK-MAPK and PI3K-AKT-mTOR, leading to cell proliferation [36, 37]. Wild-type EGFR signaling contributes to tumor cell proliferation, evasion of apoptosis, angiogenesis and metastasis [38, 39]. In recent years, some studies revealed the crosstalk between EGFR and LPS-TLR4 signal pathway. Koff et al showed that LPS induced EGFR activation via TLR4/TNF-α converting enzyme pathway [22, 40]. And Küper et.al found that in renal medullary collecting duct cells bingding of LPS to the TLR4 receptor induces activation of EGFR and MAPKs [41]. Although EGFR is regularly expressed in cardiomyocytes and cardiac derived TNF-α is mainly involved in promoting cardiovascular failure in endotoxemia or sepsis. There is no study demonstrating the effect of EGFR on the production of myocardial TNF-α in endotoxemia or sepsis. Here, we demonstrated that both EGFR irreversible and reversible inhibitor PD168393 or Erlotinib can effectively inhibit the production of TNF-α in cardiomyocytes after LPS treatment, especially for PD168393, which decreases the production of TNF-α in a dose dependent-manner. Although both PD168393 and erlotinib are selective inhibitors of EGFR, according to Fabian et al's study, this kind of inhibition is still lacking of specificity [42]. So, si-EGFR was applied to specifically knock down the expression of EGFR. We also found that the inhibition of EGFR expression was associated with decreased TNF-α mRNA and protein levels. EGFR can activate downstream PI3K/AKT and MAPK for cell proliferation and MAPK is the most important signal molecular regulating the generation of TNF-α in endotoxemia or sepsis [43, 44]. In this study, we proved that EGFR activation increases the generation of TNF-α via promoting p38 and ERK1/2 phosphorylation.

TACE is reported to be responsible for the ectodomain shedding of TGF-α in various epithelial tissues [25, 45]. Shao MX et al. found that TACE activity is required for the phosphorylation of EGFR in the production of MUC5AC mucin induced by LPS in airway epithelial cells [46]. Therefore TAPI-1 was used to inhibit the activities of TACE in cardiomyocytes. In this circumstance, LPS could not transactivate EGFR and increase the expression of TNF-α mRNA. As TACE activity is also responsible for the shedding of TNF-α protein, so the inhibitory effect of TAPI-1 on the expression of TNF-α mRNA is mainly due to the decrease of EGFR phosphorylation. LPS increases the amount of TGF-α in the medium of neonatal cardiomyocytes *in vitro*. Subsequently,*in vivo* TGF-α protein staining indicated that the increased TGF-α in response to LPS derived from cardiomyocytes. TGF-α, released by TACE, is a ligand for EGFR. This can explain how LPS transactivates EGFR through TACE/TGF-α, which was further proved by TGF-α neutralizing antibodies. On the other hand, in our experiments, exogenous TGF-α protein could not only increase the expression of TNF-α mRNA but also alleviate the inhibitory effect of TAPI-1 on TNF-α mRNA expression in response to LPS. All these results indicated a critical role of TGF-α in the trans-activation of EGFR induced by LPS in cardiomyocytes. Whether TGF-α can serve as a new target for the treatment of sepsis or endotoxemia needs to be further studied.

As TNF-α is a important cytokine responsible for cardiac damage and dysfunction during sepsis or endotoxemia and cardiovascular failure is the major reason for the death of septic shock [6, 47]. Therefore, cardiac ultrasound was applied to measure the pump function of left ventricle 6 hours after LPS treatment with or without erlotinib treatment. The results were very exciting that erlotinib either pretreatment or treated at the same time with LPS could dramatically reverse LPS induced cardiac dysfunction in endotoxemic mice. To avoid systemic reflex influences, 6h after *in vivo* LPS treatment mice hearts were isolated to assess cardiac function more directly through ligandorff system. We also got the similar results as echocardiography. Therefore, this may be one of the major reasons that erlotinib before or after LPS stimulation can significantly improve survival rate during acute endotoxemia in mice. Some other studies also reported that in LPS treatment, inhibiting the phosphorylation of EGFR could decrease the expression of COX-2 and some inflammatory cytokines such as IL-1 and IL-6 [23, 40]. These may also contributed to erlotinib's protective effect on the heart of endotoxemic mice. All these results indicated a potential new treatment target and the application of erlotinib in sepsis.

## MATERIALS AND METHODS

### Materials

Erlotinib was from Selleck (USA). PD168393 was from EMD Chemicals, Inc (San Diego, CA, USA). Liberase TH was from Roche (Mannheim, Germany). Mouse TNF-α and TGF-α ELISA kit were from eBioscience (San Diego, CA, USA). TGF-α neutralizing antibody, EGFR neutralizing antibody and TAPI-1 were from Calbiochem (Germany). Quantitative real-time PCR mix buffer was from Promega (Madison, WI, USA). TRIzol and all culture medium and supplements were from Gibco (USA). LPS was from Sigma (Oakville, Ontario Canada). Oligo and Lipo2000 was from GenePharma (Shanghai GenePharma Co., Ltd). TGF-α was from Peprotech (USA). TGF-α antibody was from Biosynthesis (Beijing China)

### Animals

Experimental protocols were approved by the local council of ethics and performed in accordance with the Guidelines for the Care and Use of Laboratory Animals of Nanfang Hospital. To in accordance with the guidelines of the International Association for the Study of Pain as published in Pain 1983; 16:109-110, all the operation were done, after animals were anesthetized with urethane, so animals did not feel pain or discomfort during the experiments and the minimum possible pain or stress had been imposed on the animals. Eight-week-old C57BL/6 mice were adopted from the experimental animal center of Southern Medical University with a mean body weight of 25 g. Survival was obtained in the following five groups of mice: (1) control group-received intraperitoneal (i.p.) injections of saline; (2) Erlotinib group-received erlotinib pretreatment for 3 days (45 mg/kg, p.o. 3d); (3) LPS group received LPS (20 mg/kg, i.p.); (4)LPS + erlotinib (45 mg/kg p.o. 3d) group-received erlotinib pretreatment orally for 3 days followed by LPS (20 mg/kg i.p.); (5) LPS + erlotinib (45 mg/kg i.p.) group-received erlotinib once by intraperitoneal injection the same time with LPS (20 mg/kg i.p.)

### Preparation of neonatal mouse cardiomyocytes

Neonatal hearts from C57BL6 mice born within 24h were minced in a nominally Ca^2+^ and Mg^2+^ free D-Hanks balance solution. Cardiac myocytes were dispersed by the addition of Liberase TH with a final concentration of 22.5 μg/mL in D-Hanks solution and incubated in 37°C water bath for 10 min. After being mixed by pipette for about 2-4 min, the supernatant was collected into a 15ml clean tube. Then adding new fresh digestion buffer and cell suspension was centrifuged at 800 rpm for 5 min to obtain a cell pellet and the debris of heart was redigested one more time by Liberase TH and collected in the same tube. Cells were then suspended in M199 medium supplemented with 10% fetal bovine serum (FBS) and 1% penicillin-streptomycin solution and preplated for 45-60 min to remove noncardiomyocytes. Then the cell suspension was filtered through a polypropylene macroinvolved porous filter (mesh opening 105 μm, Spectra/Mesh, Spectrum Medical Industries). The cardiomyocytes were plated at a density of 5×10^5^ cells/ml in M199 supplemented with 10% FBS and 1% penicillin-streptomycin solution on 24-well plates precoated with 1% gelatin. Cells were incubated at 37°C in a humidified atmosphere containing 5% CO_2_. A confluent monolayer of spontaneously beating cells was formed within 2 days.

### EGFR siRNA preparation and transfection

The EGFR-specific siRNA duplexes oligo(Shanghai GenePharma Co., Ltd) was purchased. The sequences of oligo are (sense) 5′-CUCCAGAGGAUGUUCAAUATT-3′ and (antisense) 5′-UAUUGAACAUCCUCUGGAGTT-3′. We use Lipo2000(Shanghai GenePharma Co., Ltd) as a nonspecific siRNA control. siRNA was transfected into myocardium. RT-qPCR was used to dectect EGFR silencing by siRNA after transfection.

### RT-qPCR

Total cellular RNA was isolated by using Trizol reagent. cDNA was synthesized using total RNA (1μg) and SuperScript reverse transcriptase (PrimeScript ^TM^reagent Kit, TaKaRa) according to standard protocols. Amplification was performed with the default PCR setting:denatured at 95°C for 30 sec, followed by 40 cycles of 95°C for 30 sec. and of 55°C for 30 sec. and 72°C for 30 sec. Using a SYBR Premix Ex Taq II. The following primers were used:EGFR:forward 5′-AACTGTGAGGTGGTCCTTGG-3′, reverse 5′-GTTGAGGGCAATGAGGACAT-3′;TNF-α: forward 5′-CCCCAAAGGGATGAGAAGTT-3′, reverse 5′-CACTTGGTGGTTTGCTACGA-3′ ;GAPDH: forward 5′-AACTTTGGCATTGTGGAAGG-3′, reverse 5′-GGATGCAGGGATGATGTTCT-3′. Relative quantification of gene expression was determined by using the comparative Ct method.

### Western blot analysis

Total proteins were extracted from the neonatal cardiomyocytes or myocardium with the lysis buffer supplemented with 1mM PMSF, and a protease inhibitor cocktail. Protein in the supernatant was quantified using a BCA protein assay kit (Beyotime Institute of Biotechnology, China). 20 to 50 μg of protein in each sample was subjected to polyvinlidene difluoride (PVDF) membranes. Blots were probed with specific antibodies against EGFR, phospho-EGFR (Y1068), ERK, phospho-ERK(1:1500), p38, phospho-p38(1:1000, Cell Signaling Technology, Danvers, MA, USA) respectively. Horseradish peroxidase-conjugated anti-rabbit immunoglobulin G (1;2000; Cell Signaling Technology) was used as secondary antibody. The membranes were examined with a Kodak image station 2000R apparatus (Kodak, Rochester, NY, USA). β-actin was used as the control for equal loading of the protein.

### Measurement the concentration of erlotinib in the plasma of mice

Fourty C57BL/6 mice (male, 20-30g) were randomly divided into two groups: erlotinib (45 mg/kg p.o. 3d) group and erlotinib (45 mg/kg i.p.) group. Mice blood samples were collected 0.5, 1, 2, 4, 6 and 12 h post-dose. The blood samples were centrifuged at 10 000 g for 10 min and the supernatant (plasma) was collected. The plasma 90 ul were mixed with 350 μl methanol then add 10 μl grfitinib (20 μg/ml, as the internal standard), followed by vortex and centrifugation (15 min, 13000 g), The supernatant was collected and dried, then redissolve by 200 μl 50% acetonitrile-water, followed by vortex, sonicated (10 min) and centrifuged at 13000 g (10 min), A 20 μl aliquot of the supernatant was subjected to HPLC analysis. The separation was performed using the Agilent 1260 HPLC system. Chromatographic elution was performed on the 5C18-MS-II column (20-250mm, Cosmosil) using an isocratic gradient of 35% acetonitrile in water. The detection wavelength was at 210 nm.

### *In vivo* TGF-α positive score

For the cases with weak staining in most sights, we chose five sights in random. For the cases with strong staining, we selected one sight in random for scoring. Each 20X field had 200 cells available for analyzing at least. The immunostaining results were scored by the proportion of cells staining positive as follows: 0 for 1%-5% of cells, 1 for 6-25% of cells, 2 for 26-50% of cells, 3 for 51-75% of cells and 4 for >75%; staining intensity of cancer cells was graded as 0 (no staining), 1 (weak staining, light yellow), 2 (moderate staining, yellowish brown) and 3 (strong staining, brown). The histological score (H-score) of the tissue for each section was computed by the following formula: H-score = ratio score + intensity score. A total score of 0-1 was graded as negative (−, score 0-1), weak (+, score 2-3), moderate (++, score 4-5) or strong (+++, score 6-7) for further nonparametric testing. Among them, the staining level negative and weak was considered as low expression, whereas moderate and strong was regarded as overexpression

### Measurement of TNF-α and TGF-α

TNF-α and TGF-α protein were measured with a mouse TNF-α and TGF-α ELISA kit (eBioscience, San Diego, CA, USA), according to the manufacturer's instructions. The measurements were standardized with cell numbers. Total RNA was extracted from cardiomyocytes with TriZol reagent (Gibco) according to the manufacturer's instructions.

### Echocardiography

Adult male C57BL/6 mice (8-weeks old) were randomly divided into five groups. (1) control group-received intraperitoneal (i.p.) injections of saline; (2) Erlotinib (45 mg/kg p.o. 3d); (3) LPS (20mg/kg, i.p.); (4) LPS + erlotinib (45 mg/kg, po 3d) group; (5) LPS + erlotinib (45 mg/kg i.p.) group. After 6 h, mice were anaesthetized with 0.5-1% halothane inhalation in a mixture of 95% O_2_ and 5% CO_2_. Echocardiography (Visual Sonic, Vevo2100) was performed. A 30 MHz probe (Visual Sonic, Vevo2100) placed in the parasternal, short-axis orientation recorded LV systolic (LVIDs) and diastolic internal dimensions (LVIDd). Three loops of M-mode data were captured for each animal, and data were averaged from at least 5 beat cycles. These parameters allowed the determination of left ventricular (LV) fractional shortening (FS) by the equation :FS=[(LVIDd-LVIDs)/LVIDd]×100%. Ascending aortic flow waveforms were recorded using a continuous wave Doppler flow probe oriented in a short-axis, suprasternal manner. Peak aortic flow and velocity-time integrals (VTI) were calculated from these waveforms. Cardiac output (CO) was calculated by the equation: CO=heart rate ×VTI×aortic cross-sectional area. Autopsy measurements of aortic root cross-sectional area were conducted. LV diastolic function was evaluated by the ration of the E/A wave from the transmitral valve flow waveform.

### Isolated heart preparations

Adult male C57BL/6 mice (8-weeks old) were randomly divided into four groups. (1) control group; (2) LPS group (20mg/kg, i.p.); (3) LPS + erlotinib (45 mg/kg, p.o 3d) group; (4) LPS + erlotinib (45 mg/kg i.p.) group. After 6 h of LPS treatment, hearts were isolated and the aorta was cannulated using a 20g steel cannula. Hearts were perfumed in a Langendorff-system with warm (37°C) Krebs buffer containing (in mM) 118 NaCl, 4.7 KCl, 25 NaHCO3, 1.2 MgSO4, 1.2 KH2PO4, 2 CaCl2 gassed with 95% O2, 5% CO2. Hearts are perfused with glucose 11mM as sole substrate or in combination with 1 or 1.2 mM palmitate. The pulmonary artery is transected to facilitate coronary venous drainage. A left ventricular polyethylene apical drain is inserted through a left atrial incision to allow thebesian venous drainage. Left ventricular pressure is monitored from a water-filled balloon placed through the left atrial appendage and connected to a Millar transducer. The heart work was calculated by multiplying the force (g) by the heart rate (bpm). Maximal and minimal first derivatives of force (+dF/dtmax and -dF/dtmin) as the rate of contraction and relaxation were analyzed by PowerLab Chart program (AD Instruments).

### Statistical analysis

Results are presented as mean±SD. Differences between two groups were analyzed by a Student t test. For multi-group comparisions, One-way ANOVA was performed. Survival studies were analyzed by Chi-square test. *P* < 0.05 was considered statistically significant. Graphs and figures were made with Graphpad Prism 6 (GraphPad software, CA, USA).
